# 

**Published:** 1898-01

**Authors:** 


### CONTENTS.

ORIGINAL COMMUNICATIONS. PAGE

A Study of the Deformities of the Jaws among the Degenerate Classes of Europe. By EUGENE S. TALBOT, M.D., D.D.S. . . . . 1

Spring Levers for regulating Teeth. By W. S. DAVENPORT, Paris, France . . . 9

The Therapeutic Treatment of Infected Tooth-Root Canals. By GEORGE W. WARREN, D.D.S. . . . . 11

Formaldehyde in Solid Form. By WILLIAM ROLLINS, Boston, MASS. . . . . 15

ABSTRACTS AND TRANSLATIONS.

The Preparation and Coloring of Lantern-Slides suitable for Scientific Demonstrations. By J. ALFRED SCOTT, M.A., M.D., F.R.C.S.I. . . . . 15

REPORTS OF SOCIETY MEETINGS.

American Dental Association . . . . 19

Joint Convention of the American and Southern Dental Associations . . . . 45

American Academy of Dental Science . . . . 48

Odontological Society of Pennsylvania . . . . 53

EDITORIAL.

Wrong Impressions . . . . 60

BIBLIOGRAPHY . . . . 62

OBITUARY.

Chicago Dental Society—Resolutions of Respect . . . . 64

